# Quantification of Acute Vocal Fold Epithelial Surface Damage with Increasing Time and Magnitude Doses of Vibration Exposure

**DOI:** 10.1371/journal.pone.0091615

**Published:** 2014-03-13

**Authors:** Tsuyoshi Kojima, Mark Van Deusen, W. Gray Jerome, C. Gaelyn Garrett, M. Preeti Sivasankar, Carolyn K. Novaleski, Bernard Rousseau

**Affiliations:** 1 Department of Otolaryngology, Vanderbilt University School of Medicine, Nashville, Tennessee, United States of America; 2 Department of Hearing and Speech Sciences, Vanderbilt University School of Medicine, Nashville, Tennessee, United States of America; 3 Department of Pathology, Microbiology and Immunology, Vanderbilt University School of Medicine, Nashville, Tennessee, United States of America; 4 Department of Speech, Language, and Hearing Sciences, Purdue University, West Lafayette, Indiana, United States of America; Federal University of Rio de Janeiro, Brazil

## Abstract

Because the vocal folds undergo repeated trauma during continuous cycles of vibration, the epithelium is routinely susceptible to damage during phonation. Excessive and prolonged vibration exposure is considered a significant predisposing factor in the development of vocal fold pathology. The purpose of the present study was to quantify the extent of epithelial surface damage following increased time and magnitude doses of vibration exposure using an *in vivo* rabbit phonation model. Forty-five New Zealand white breeder rabbits were randomized to nine groups and received varying phonation time-doses (30, 60, or 120 minutes) and magnitude-doses (control, modal intensity phonation, or raised intensity phonation) of vibration exposure. Scanning electron microscopy and transmission electron microscopy was used to quantify the degree of epithelial surface damage. Results revealed a significant reduction in microprojection density, microprojection height, and depth of the epithelial surface with increasing time and phonation magnitudes doses, signifying increased epithelial surface damage risk with excessive and prolonged vibration exposure. Destruction to the epithelial cell surface may provide significant insight into the disruption of cell function following prolonged vibration exposure. One important goal achieved in the present study was the quantification of epithelial surface damage using objective imaging criteria. These data provide an important foundation for future studies of long-term tissue recovery from excessive and prolonged vibration exposure.

## Introduction

The vocal folds are a pair of connective tissues located within the larynx, the primary organ involved in voice production. To create voice, the vocal folds modulate airflow from the lungs. The act of voice production is often referred to as phonation. The vocal folds undergo continuous and repeated cycles of trauma during phonation. Vocal fold trauma, more commonly referred to as phonotrauma, results from prolonged and excessive vibration exposure. Phonotrauma is widely believed to contribute to alterations to the tissue’s native microarchitecture. Morphological and structural changes may disrupt tissue vibration and lead to the development of vocal fold pathologies. Clinically, phonotrauma can present as hemorrhage, sub-epithelial edema, and nodular formations of the vocal folds. It has been estimated that approximately 30% of adults in the United States will be affected by a voice disorder at some point during an individual’s lifespan [1], [2]. The estimated direct costs associated with the assessment and treatment of voice disorders in the United States is upwards of $11.9–13.5 billion annually, resulting in a considerable cost to society and a significant impact on an individual’s emotional, functional, and physical well-being [3]–[5].

Histologically, the vocal folds consist of several layers, including epithelium, lamina propria, and muscle [6]. The outer tissue layer of the vocal folds is a stratified squamous epithelium and among its important functions is to protect the underlying connective tissue. The integrity of the vocal fold’s layers is important for tissue viscoelasticity and normal voice production. The epithelium has a physical protective barrier that is comprised of proteins referred to as the junctional complex. Although previously believed to serve as a passive barrier to environmental and mechanical stresses, the vocal fold epithelium has emerged as an important cell layer involved in critical functions such as active ion transport and maintenance of surface fluid composition [7], [8]. The epithelial barrier acts as a critical boundary that defends against environmental and other stresses that have the potential to harm the vocal folds.

The vocal fold epithelial cells are covered with dense microprojections [9]. Microprojections are also found on the epithelium of the cornea, intestines, and kidney glomerular [10]–[12]. The structure and function of microprojections in other epithelial tissues has been well described. It has been suggested that corneal microprojections enlarge the cell surface area to facilitate nutrient and oxygen absorption, stabilize secretions, and assist with water and metabolic product movement across outer epithelial cell membranes [13], [14]. In comparison to corneal microprojections, the function of vocal fold epithelial microprojections is less understood. However, it has been speculated that surface microprojections on the vocal folds are involved in water absorption, mucus adherence, and traction during normal phonation [15]. In addition, they may help lubricate the epithelium by distributing and retaining mucus [9], [15]. Moreover, it is possible that microprojections may serve as an important protective epithelial barrier against external stimuli by assisting with homeostasis of normal tissue function.

Considering the magnitude of tensile, shear, and impact stresses that the vocal folds are exposed to during vibration, the tissue’s unique composition may serve an important role in the protection of the vocal folds from injury [16]. The estimated magnitude of biomechanical stresses during phonation for males is 10–100 Pa at lower frequencies and 40–1000 Pa at higher frequencies [17]. It is believed that the most significant contributor to epithelial damage is impact stress [16]. We hypothesize that objective changes in microprojection integrity may provide significant insight into the pathophysiology of impaired tissue function and the susceptibility of the vocal folds to injury. The disruption of epithelial barrier integrity may compromise important physiologic mechanisms that are critical to normal tissue function. Moreover, the vocal folds provide a unique system in the study of epithelial barrier response to various challenges. The effects of prolonged phonation and transient episodes of phonotrauma have been described by our group and others [18], [19]. Previously, Gray and Titze reported desquamation of epithelial cells and basement membrane injury after long durations of phonation in canines [18]. More recently, our group developed an *in vivo* rabbit phonation model to examine the cellular and molecular events underlying acute phonotrauma. Our studies have revealed alterations to epithelial surface morphology, dilation of epithelial tight junctions, microhole formation, and gene expression changes following transient episodes of phonotrauma [19]. While these studies have confirmed the potential of epithelial surface damage risk, no investigations to date have attempted to quantify the amount of epithelial surface damage resulting from phonotrauma.

Anecdotally, prolonged and excessive vibration exposure is often cited as one of the leading causes of vocal fold pathology. Because the vocal folds undergo continuous and repeated cycles of trauma during phonation, the tissue is exposed to considerable mechanical stress. These perturbations present a significant challenge to the epithelial barrier. Significant damage to the vocal fold epithelium has been reported following transient and prolonged episodes of vibration exposure [18], [19]. What remains unknown, however, is the effect of increasing time and magnitude doses of vibration exposure on vocal fold barrier integrity. This requires the application of objective approaches to precisely measure vocal fold epithelial surface changes and the use of an animal model suitable for studies of long-term tissue recovery. As a logical and necessary first step, the purpose of the present study was to quantify the effects of increasing time-doses and magnitude-doses (i.e., modal intensity phonation, raised intensity phonation) of vibration exposure on key cell surface features using an *in vivo* rabbit phonation model. Gray and Titze [18] proposed that microprojection destruction is one of the early signs of injury to the cell surface. Therefore, in the current study we focused on examining damage to the epithelial cell surface to quantify these early signs of tissue injury. Scanning electron microscopy (SEM) and transmission electron microscopy (TEM) under high-powered field along with advanced measurement techniques were used to evaluate the epithelial surface microprojections and surface layers of the vocal folds.

## Materials and Methods

### Animals

The procedures used in this study were performed in accordance with the Public Health Service Policy on Humane Care and Use of Laboratory Animals, National Institutes of Health Guide for the Care and Use of Laboratory Animals, and Animal Welfare Act (7 U.S.C. et seq.). The animal use protocol was approved by the Institutional Animal Care and Use Committee of Vanderbilt University Medical Center. This study used 45 New Zealand white breeder rabbits that weighed between 2.8–3.8 kg. Rabbits were anesthetized through intramuscular administration of ketamine (35 mg/kg), xylazine (5 mg/kg), and acepromazine (0.75 mg/kg). To assess the safety and wellbeing of the animals, oxygen saturation level, temperature, and heart rate were continually monitored. To maintain the effects of anesthesia, ketamine (17.5 mg/kg) and acepromazine (0.375 mg/kg) were injected intramuscularly as needed. A between groups research design was used to objectively measure the effects of varying time- and magnitude-doses of vibration exposure on vocal fold epithelial surface damage. Animals were randomized to nine groups (*n* = 5) that received 30, 60, or 120 minutes of vocal fold approximation without phonation (control condition), modal intensity phonation, or raised intensity phonation. Intensity levels and phonation times were selected based on previous experiments conducted in our laboratory, which have demonstrated that airflow rates of approximately 144 cm3/sec in combination with stimulation currents of 0.2–0.6 mA above those necessary to produce modal intensity phonation produce phonations that are 5–10 dB louder than modal intensity and are associated with changes to inflammatory mediator gene expression and alterations to the integrity of the vocal fold epithelial barrier [19]–[22].

### Phonation Procedures

To prepare for surgery, all rabbits were placed on an operating platform in the supine position and shaved at the neck from the submentum down to the chest. As described previously [19], a midline incision from the hyoid bone to the sternal notch was used to expose the larynx and trachea. The trachea was transected proximal to the sternum. Sutures were placed to suspend the lower portion of the trachea to the sternal fascia, providing a stable airway via a tracheostomy. A cuffed endotracheal tube (Mallinckrodt, Hennef, Germany) (3.5 mm) was then inserted into the upper portion of the bisected trachea and placed 2 cm below the opening of the glottis. To close off the trachea and deliver airflow through the glottal opening, the endotracheal tube cuff was inflated.

Stainless-steel hooked electrodes were used to deliver electrical stimulation. One electrode was inserted into the belly of each cricothyroid muscle perpendicular to the muscle fibers (cathodes) and one electrode was inserted into the cricothyroid membrane on each side at the intersection of a longitudinal line 1 mm lateral to midline and a transverse line 1 mm inferior to the thyroid cartilage (anodes). Compressed, humidified air at 37°C was delivered to the glottis using a Gilmont Instruments flowmeter (GF-8522-1700; Barrington, IL) and Concha Therm Neptune humidifier (Hudson, RCI, Temecula, CA). To induce electrical stimulation to the laryngeal apparatus, a Grass S-88 stimulator (SA Instrumentation, Encinitas, CA) and constant current isolation unit (Grass Telefactor, model PSIU6; West Warwick, RI) were used. The total train duration was 10 seconds with 3 seconds of electrical stimulation and 7 seconds of rest [20]–[22]. To confirm proper vocal fold positioning, video imaging of the vocal folds was captured using a 30° 2.7-mm rigid endoscope (Karl Storz Endoscopy- America, Inc., El Segundo, CA) and Telecam-C camera (Karl Storz Endoscopy-America, Inc.).

**Figure 1:**
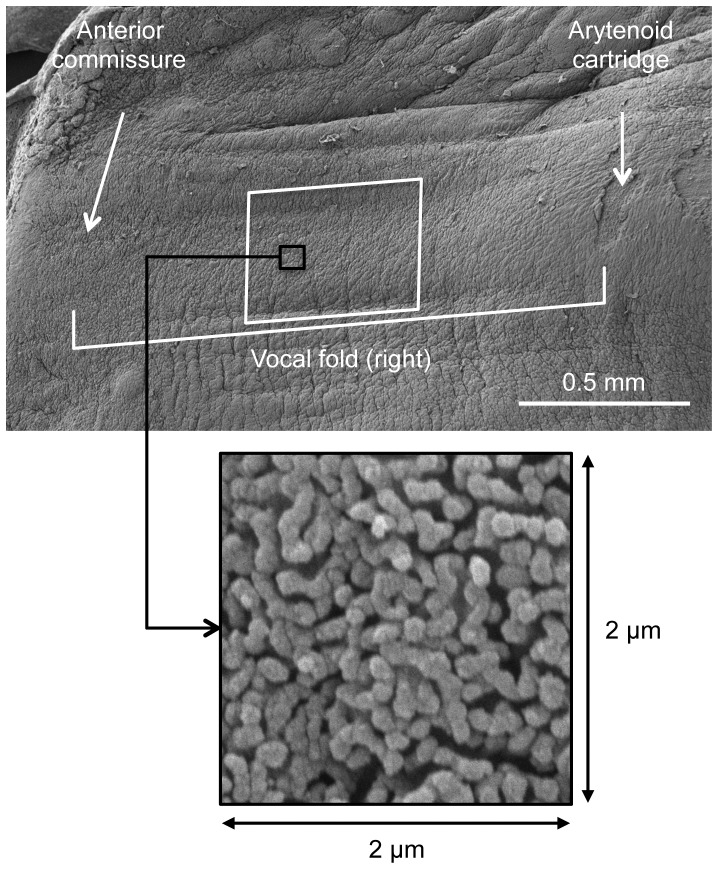
SEM image of a rabbit vocal fold. Scanning electron microscopy image of a rabbit vocal fold. Large box represents the central portion of the middle one-third region of the vocal fold. Small box represents a 2×2 µm image used for SEM analysis.

Acoustic measures were collected during the procedures to detect changes in phonation intensity and mean fundamental frequency. Acoustic signals were recorded at baseline and in 15-minute intervals throughout the phonation procedure using a Perception 170 Condenser Microphone (AKG, Vienna, Austria) that was positioned 10 cm from the opening of the laryngoscope. Mouth-to-microphone distance and amplitude gain were held constant throughout the audio recordings. Recordings were digitized using Computerized Speech Lab Model 4500 (KayPENTAX, Montvale, NJ) using the system’s factory calibrated settings. The most stable 0.5- to 1.0-second portion of the acoustic waveform was selected and analyzed to measure mean phonation intensity and mean fundamental frequency. Acoustic analyses revealed that during modal intensity phonation, mean vocal intensity was 60.56 dB (SD = 6.03) at baseline and was subsequently maintained at 61.41 dB (SD = 3.76) during subsequent time intervals. In contrast, during raised intensity phonation, mean vocal intensity was 65.84 dB (SD = 7.37) at baseline and subsequently maintained at 67.69 dB (SD = 4.66). Throughout the experiment, mean fundamental frequency was maintained at 665.39 Hz (SD = 151.12) during modal intensity phonation and 773.79 Hz (SD = 175.52) during raised intensity phonation. It should be noted that prolonged rabbit phonation may not be a direct physiologic representation of vocal fold vibration in humans. The fundamental frequency of rabbit phonation is approximately three times the frequency of human phonation, suggesting that rabbits may be susceptible to more frequent episodes of vocal fold collision. However, because rabbit vocal folds are shorter and have less mass than human vocal folds, it may be possible that the smaller amplitudes and impact stresses offset the higher fundamental frequencies to some degree. Although there are known differences between rabbit and human laryngeal anatomy, animal models provide the unique ability to investigate tissue molecular and structural alterations, which require harvesting of the vocal folds. Following each procedure, rabbits were euthanized and larynges were harvested 30 minutes after the end of the phonation experiment. Harvested tissue was soaked in 2.5% Glutaraldehyde solution and stored at 4° centrifuge after being at room temperature for one hour. One vocal fold was used for SEM and the contralateral vocal fold was used for TEM. All analyses were performed using the central portion of the middle one-third region of the vocal fold (Figure 1).

### Scanning Electron Microscopy Procedures

**Figure 2:**
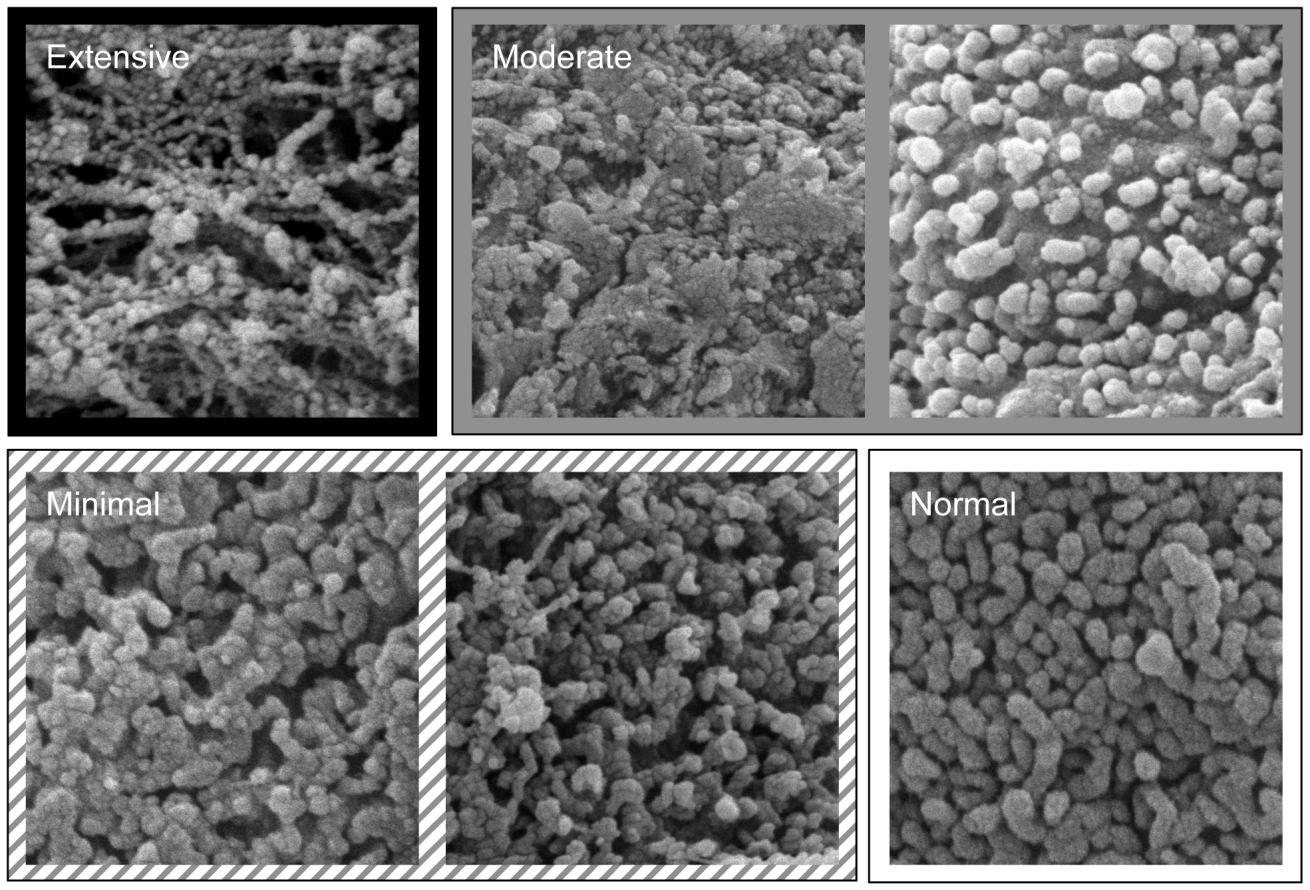
Damage severity descriptions used for visual evaluation of microprojections. All images were categorized into four groups according to the degree of surface damage. (1) extensive – the cell surface was destroyed and cytoskeleton was exposed; (2) moderate – all microprojections were damaged and appeared flat or normal; (3) minimal – microprojections were slightly damaged; and (4) normal – microprojections showed no damage.

Routine methods for tissue processing for SEM were used [23], [24]. SEM images were acquired using a Quanta 250 Environmental Scanning Electron Microscope (FEI Company, Hillsboro, OR). Damage to the vocal fold epithelial surface was evaluated using both standard visual examination and objective imaging techniques. During routine visual examination, ten 2×2 µm SEM images (original magnification was 20000×) were randomly selected from each of the nine groups. The images were randomly presented to two blinded judges who used a severity scale based on four categories of damage severity: extensive, moderate, minimal, and normal (Figure 2) for classification of images into groups. Criteria for extensive damage included a destroyed cell surface and exposed cytoskeleton. Moderate damage included flattening or denting of all microprojections, or the removal of normal cells and emergence of newer cells. Minimal damage was characterized by slightly dented microprojections, while the normal category displayed no microprojection damage. After classification of images into groups, the two judges completed a consensus rating to reclassify images in which there were discrepancies.

**Figure 3:**
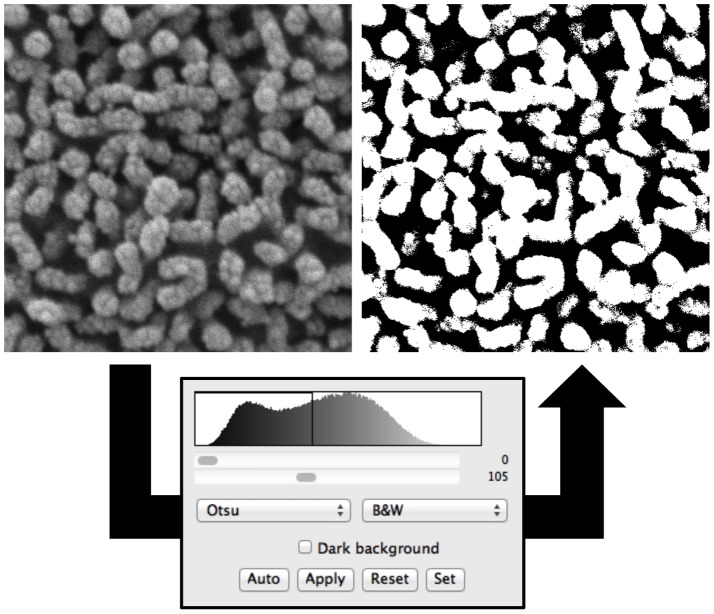
Otsu’s Method. 2×2 µm original image (left) and binary image (right) generated using ImageJ demonstrating the objective evaluation of vocal fold epithelial surface microprojection density using Otsu’s method.

To evaluate SEM images using objective criteria, Otsu’s method was used, which is based on a binary imaging algorithm [25], [26]. The goal of Otsu’s method, which is founded on image histograms, is to objectively convert grayscale images to black and white images. The pixel variance was calculated as two separate groups in which: 1) pixels are at or below all possible thresholds, and 2) pixels are above all possible thresholds. An optimal threshold value was then selected, which separates the two groups to minimize the intra-class variance. Intra-class variance is defined as the sum of the two variances multiplied by their associated weights. Maximizing inter-class variance produces the same optimal threshold. The selected threshold was specific to each individual image, but calculated in an automatic and consistent manner. Every image pixel was quantitatively categorized as either an image object or image background according to the optimal threshold using ImageJ software (W. Rasband, National Institutes of Health, Bethesda, MD) [27]. In this study, microprojections were represented as white pixels, which were clearly distinguished from the background that was represented as black pixels (Figure 3).

### Transmission Electron Microscopy Procedures

Routine methods were used for tissue processing for TEM [23], [24]. TEM images were acquired using a Philips CM-12 transmission electron microscope (FEI Company, Hillsboro, OR). Epithelial damage was quantified via ratings of three parameters of epithelial surface microprojections using TEM images: microprojection density, microprojection height, and depth of the remaining cell surface. For every tissue sample (*N* = 45), five randomized regions were selected for examination using ImageJ [27] and the mean value of each parameter was obtained. Microprojection density was defined as the number of microprojections within a distance of 10 µm. This was calculated by counting five consecutive microprojections and measuring the distance of these five consecutive microprojections using original magnification 15000–53000×. A calculation was then performed in which 5 was divided by the distance of five consecutive microprojections and multiplied by 10. Microprojection height was measured as the entire length of the microprojection using original magnification 4400–8800×. The depth of the remaining cell layer was measured from the surface of the cell to the basal membrane using original magnification 4400–8800×. The viable surface cell was differentiated by a darker shade of color and the desquamated surface cell was a lighter color.

### Statistical Analysis

For each dependent variable, Kruskal-Wallis non-parametric statistical testing was used to investigate for overall main effects across all conditions. Alpha levels were adjusted to 0.0125 to control for type I error (0.05/4) using two-tailed *p* values. If the overall main effect was significant, post-hoc pairwise comparisons were performed using Mann-Whitney *U* tests for two independent samples to investigate for differences between conditions. Alpha levels were adjusted to 0.0167 to control for type I error (0.05/3) using two-tailed *p* values. A Pearson *r* correlation coefficient was computed to determine the level of agreement between microprojection density measured by SEM and TEM. All data were analyzed using IBM SPSS 21.0 (International Business Machines Corp., Armonk, NY).

## Results

### Scanning Electron Microscopy

#### Visual Examination of Epithelial Surface Microprojections

**Figure 4:**
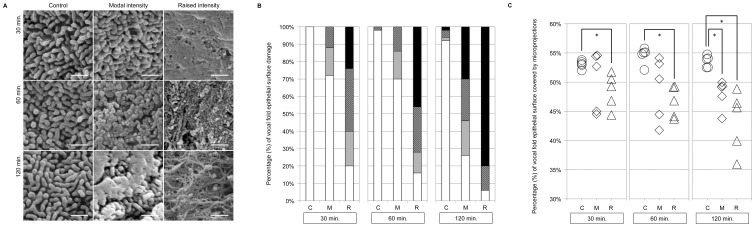
A. SEM representative images for control, modal intensity, and raised intensity phonation. Representative 2×2 µm SEM images for the control, modal intensity, and raised intensity phonation conditions after 30, 60, and 120 minutes of phonation. Measurement bar represents 0.5 µm. B. Visual examination of epithelial surface microprojections. Classification of images by damage severity using routine visual examination of epithelial surface microprojections. Four categories of damage severity: extensive (black fill), moderate (dark gray fill), minimal (light gray fill), and normal (white fill) in the control (C), modal intensity phonation (M), and raised intensity phonation (R) conditions after 30, 60, and 120 minutes of phonation. C. Objective examination of epithelial surface microprojection density using SEM. The percentage of vocal fold surface covered by microprojections in the control (C), modal intensity phonation (M), and raised intensity phonation (R) conditions after 30, 60, and 120 minutes of phonation. * Denotes a significant difference between groups (p<0.01).

Visual examination of vocal fold epithelial surface microprojections revealed extensive damage to the epithelial surface and decreased density of epithelial surface microprojections with increasing time- and magnitude-doses of vibration exposure, (Figure 4-A). In contrast, control vocal folds matched at each time and magnitude dose revealed limited to no damage. After 30 and 60 minutes of modal intensity phonation, minimal to moderate damage was observed on less than 30% of the cell surface (Figure 4-B). After 120 minutes of modal intensity phonation, minimal to extensive damage was observed on greater than 70% of the cell surface area, with a relatively equal distribution of minimal, moderate, and extensive damage observed across all vocal fold samples (Figure 4-B). A similar pattern was observed after 30 and 60 minutes of raised intensity phonation. However, more extensive damage was present after only 30 minutes of raised intensity phonation, in comparison to the same degree of damage evident following 60 minutes of modal intensity phonation. After 120 minutes of raised intensity phonation, extensive damage was present on greater than 80% of the vocal fold cell surface area (Figure 4-B).

#### Objective Examination of Epithelial Surface Microprojection Density

Objective measurement of vocal fold epithelial surface microprojection density using SEM revealed a significant decrease in the density of epithelial surface microprojections with increasing magnitude-doses of vibration exposure. The percentage of vocal fold surface covered by microprojections was highest in the control condition (55%) (Figure 4-C). Statistical analysis of epithelial surface microprojection density revealed a significant overall main effect (*p* = 0.001). Post-hoc testing revealed a decrease in the percentage of vocal fold surface covered by microprojections after 30 minutes (*p* = 0.008), 60 minutes (*p* = 0.008), and 120 minutes (*p* = 0.008) of raised intensity phonation, compared to the control condition (Figure 4-C); and a significant decrease in the density of epithelial surface microprojections after 120 minutes (*p* = 0.008) of modal intensity phonation, compared to control. No significant differences in the density of epithelial surface microprojections were found after 30 and 60 minutes of modal intensity phonation, compared to control.

### Transmission Electron Microscopy

#### Objective Examination of Epithelial Surface Microprojection Density

**Figure 5:**
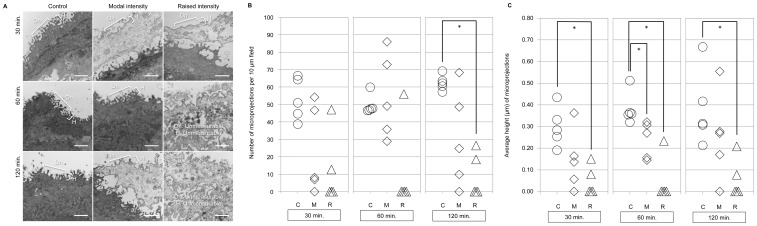
A. Measurement of epithelial surface microprojection density and height. Objective measurement of the density (Dn) and height (H) of vocal fold epithelial surface microprojections in the control (C), modal intensity phonation (M), and raised intensity phonation (R) conditions after 30, 60, and 120 minutes of phonation. Measurement bar represents 1 µm. B. Objective examination of epithelial surface microprojection density using TEM. The number of microprojections per 10 µm field in the control (C), modal intensity phonation (M), and raised intensity phonation (R) conditions after 30, 60, and 120 minutes. * Denotes a significant difference between groups (p<0.0167). C. Objective examination of epithelial surface microprojection height using TEM. Average height (µm) of microprojections in the control (C), modal intensity phonation (M), and raised intensity phonation (R) conditions after 30, 60, and 120 minutes. * Denotes a significant difference between groups (p<0.0167).

Objective measurement of vocal fold epithelial surface microprojection density using TEM revealed a significant decrease in the density of epithelial surface microprojections in the raised intensity phonation condition (Figure 5-A). Statistical analysis of epithelial surface microprojection density revealed a significant overall main effect (*p = *0.002). Post-hoc testing revealed a decrease in the density of vocal fold epithelial surface microprojections after 120 minutes of raised intensity phonation, compared to the control condition (*p* = 0.008) (Figure 5-B). Microprojection density was also measured using SEM, and there was a significant and positive relationship between the percentage of vocal fold covered by microprojections and the number of microprojections within a distance of 10 µm on TEM (*r* = 0.59, *p* = 0.000).

#### Objective Examination of Epithelial Microprojection Height

Objective measurement of vocal fold epithelial surface microprojection height using TEM revealed a significant decrease in the height of epithelial surface microprojections with increasing magnitude-doses of vibration exposure (Figure 5-C). Statistical analysis of epithelial surface microprojection height revealed a significant overall main effect (*p* = 0.001). Post-hoc testing revealed a decrease in average microprojection height after 30 minutes (*p* = 0.008), 60 minutes (*p* = 0.008), and 120 minutes (*p* = 0.008) of raised intensity phonation, compared to the control condition, and a significant decrease in microprojection height after 60 minutes of modal intensity phonation, (*p = *0.008) compared to control (Figure 5-C). It should also be noted that there were several instances in which surface microprojections were completely obliterated following raised intensity vibration exposure.

#### Objective Examination of Depth of the Remaining Cell Surface

**Figure 6:**
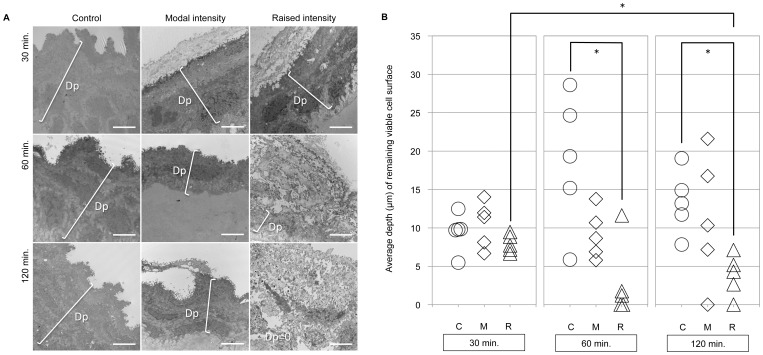
A. Measurement of depth of the remaining viable cell surface. Objective measurement of the depth (Dp) of the remaining viable cell surface layer in the control (C), modal intensity phonation (M), and raised intensity phonation (R) conditions after 30, 60, and 120 minutes of phonation. Measurement bar represents 4 µm. B. Objective examination of depth of the remaining viable cell surface using TEM. Average depth (µm) of the remaining viable cell surface in the control (C), modal intensity phonation (M), and raised intensity phonation (R) conditions for 30, 60, and 120 minutes. * Denotes a significant difference between groups (p<0.0167).

Objective measurement of depth of the remaining cell surface using TEM (Figure 6-A) revealed decreased depth of the remaining vocal fold epithelial cell layer with increasing time- and magnitude-dose of vibration exposure. TEM revealed desquamation of the vocal fold epithelial surface and loosely connected surface cell layers during modal and raised intensity phonation (Figure 6-A; light gray cell surface layer). Statistical analysis of depth of the remaining viable cell surface layer revealed a significant overall main effect (*p* = 0.007). Post-hoc testing revealed a significant decrease in depth of the remaining viable cell surface after 60 minutes (*p* = 0.016) and 120 minutes (*p* = 0.008) of raised intensity phonation, compared to the control condition; and a significant decrease in depth of the remaining viable cell surface after 120 minutes of raised intensity phonation (*p* = 0.016), compared to 30 minutes of raised intensity phonation (Figure 6-B). Similar to the aforementioned observations made regarding microprojection height and density, there were also episodes in which the depth of the remaining cell surface layer was destroyed and decreased to a value of 0.

## Discussion

Previous studies by our group and others have found that phonotrauma leads to vocal fold epithelial surface damage [18], [19]. The present investigation is the first to quantitatively evaluate the extent of vocal fold epithelial surface damage with increasing time- and magnitude-doses of vibration exposure. The vocal folds are exposed to considerable amounts of impact stress during phonation. These are thought to lead to a decrease in the integrity of the vocal folds’ protective capacity and tissue pathology. Data regarding the pathophysiology of impaired tissue function after phonotrauma are lacking. Disruption of epithelial barrier may compromise important physiologic mechanisms that are crucial for homeostasis and normal tissue functioning. There remains a significant need for further studies aimed at quantifying the effects of phonotrauma on vocal fold tissue morphology and function. We believe that these studies will provide significant insight into the pathophysiology of impaired tissue function and the susceptibility of the vocal folds to injury.

Given previous reports that one of the earliest signs of tissue injury is damage to the vocal fold cell surface, as an important and necessary first step, the overarching goal of the present study was to use advanced imaging measurement techniques to quantify damage to the vocal fold epithelial surface after phonotrauma. An important goal achieved in this study was the quantification of epithelial surface damage using objective criteria. We implemented routine visual examination and objective measurement approaches using electron microscopy techniques to quantify damage to the vocal fold epithelial surface. These methods proved to be useful in quantifying changes to the vocal fold cell surface as a function of increased time- and magnitude-doses of vibration exposure. Otsu’s method has previously been successfully used to distinguish normal microprojections from the cell surface layer in the corneal epithelium [26]. In the current study, Otsu’s method was used to quantify damage to cell surface microprojections following phonotrauma. Otsu’s method proved useful in the present study for quantification of microprojection density in cases where microprojections remained present on the cell surface. More extensive surface damage extending to the cytoskeleton and basal membrane required the use of imaging techniques capable of measuring several depths of tissue layers. For this reason, TEM imaging was also used to measure microprojection density, height, and measurement of the depth of the remaining viable cell layer. Density measures provide important information regarding the area of the cell surface area covered by microprojections. This concept is similar to the quantification of microprojection density using SEM, although TEM images provide a different view of the epithelium. In fact, a significant moderate relationship was observed between the percentage of vocal fold covered by microprojections using SEM images and the number of microprojections within a distance of 10 µm using TEM images, providing evidence of a high level of agreement between the two measures. Microprojection height provides additional insight into the distance between the cell surface and outside organisms. These objective approaches were supplemented by standard visual examination and classification of images into categories of damage. Results of the present study revealed a significant decrease in microprojection density, microprojection height, and depth of the remaining cell surface layer with increasing time and magnitudes of vibration exposure. In some cases, TEM images revealed that this damage reached the basal cell layer. Exposure of the cytoskeleton and basement membrane, as observed in the current study after long durations of raised intensity phonation, may increase vocal fold susceptibility to damage by making the basal layer and lamina propria more penetrable and vulnerable to damage from physical and noxious agents in the lumen. These observations are congruent with previous findings that acute episodes of phonotrauma may compromise vocal fold epithelial barrier integrity [19].

We hypothesize that epithelial barrier destruction may compromise physiologic mechanisms that are critical to normal tissue function. Microprojections assist with stabilizing secretions and facilitating water and metabolic product movement across the cell membrane [9], [13]–[15]. In addition, compromised integrity of this important cell layer may lead to decreased stabilization of secretions and reduced water and metabolic product movement across the outer cell membranes. Maintaining a thin layer of lubricating fluid over the cell surface is essential for promoting pliability of the vocal folds and facilitating optimal phonation oscillation pressures and normal vibration [28]. Microprojection damage may lead to deficiencies in the distribution and maintenance of vocal fold secretions, thus resulting in impaired tissue function (e.g., aperiodic vibration and abnormal voice quality).

In addition, it is important to consider the depth of damage after phonotrauma. Normal vocal fold epithelium, which is composed of stratified squamous cells, undergoes a natural process of desquamation [18]. Desquamation involves the continual and regulated renewal of the epithelium through the natural shedding of the superficial epithelial layer. By the time that the surface layer is removed, the newly exposed or viable surface cell layer is fully matured [29]. In the skin, desquamation is required to maintain the function of the epithelial barrier [30]. The role of desquamation is thought to provide initial protection against invading chemical and physical toxins because the removal of the surface layer rids the epithelium of such toxins [29]. This study revealed that the depth of the remaining viable epithelial cell layer after modal intensity phonation was similar to that of the control condition, regardless of time-dose. That is, the desquamating epithelial layer remained intact during modal intensity phonation. This may provide significant protection to the underlying basement membrane and lamina propria during continued vibration exposure by allowing sufficient time for the replenishing cell layer to reach cell maturity and replace the desquamating cell surface layer. Thus, we speculate that lower magnitudes of vibration exposure may result in a more organized and efficient cell turnover and regeneration process. It is generally believed that these phonation conditions are less traumatic to vocal fold tissue [15]. Our findings provide support for the notion that the vocal fold epithelium is a resilient tissue that may withstand the less traumatic biomechanical tissue stresses produced during lower magnitudes of vibration exposure.

In contrast, the depth of the remaining cell layer decreased (e.g., became thinner) after raised intensity phonation, which may be the result of more aggressive exfoliation of the cell surface layer during increased magnitudes of vibration exposure. The depth of the epithelial cell layer decreased after 60 and 120 minutes of raised intensity phonation, compared to control. A significant time-dose effect was also detected, with epithelial cell layer depth further decreasing from 30 to 120 minutes of raised intensity phonation. Thus, the epithelial cell layer depth findings revealed that increased time and magnitude doses of vibration exposures resulted in increased damage to the epithelial cell surface. In the corneal epithelium, deeper removal of the cell surface layer results in newly exposed or young surface cells [31]. Interestingly, it has been found that experimentally induced pathogens accumulate to a greater degree in areas of extensive damage and newly exposed surface cells, which may increase tissue susceptibility to bacterial infection [32]. These findings suggest that the removal of the vocal fold epithelial surface following increased time and magnitude doses of vibration exposure may be associated with greater risk of epithelial barrier compromise. While the present study revealed structural damage to the epithelium using electron microscopy, potential mechanisms and the functional consequences of these changes can only be speculated at this time. There is a need for additional studies to examine the integrity of the epithelial barrier and the effects of tissue alterations on functional tissue measures, such as transepithelial resistance. These studies may provide further insight into the potential link between morphological tissue changes and epithelial barrier function.

From a therapeutic (e.g., tissue recovery) perspective, vocal fold healing trajectories might yield better outcomes when integrity of the outermost cell surface layer is sound. In some instances after raised intensity phonation, damage reached deep into the basal cell membrane. The basal membrane is an important attachment, anchoring the epithelium to the lamina propria [15]. Basal cells are involved in the natural desquamation process and provide a protective sub-epithelial barrier [33]. Therefore, continued vibration exposure after the destruction of the basement membrane may place the tissue at higher risk for inflammation, infection, and the development of vocal fold pathology. In these cases, it would appear sensible to allow for recovery of the regenerating epithelium before exposing the tissue to additional potentially injurious vibration exposure. Clinically, it is often stated that vocal fold lesions arise from talking too much, too often, and too loud. Thus, pacing of voice use is often used clinically to minimize trauma to the tissue. We hypothesize that damage after acute phonotrauma and continued vibration exposure may result in delayed epithelial recovery. This may be an important mechanism in the pathophysiology of vocal fold pathology, as the replenishing stratified squamous epithelial cells are neither mature enough (i.e., integrity compromised) nor able to replace the desquamating cell layers rapidly enough to protect the basement membrane and underlying lamina propria from injury.

What remains unknown, however, is the natural sequence and timing of epithelial recovery after phonotrauma. An important next-step will be to investigate the long-term recovery of epithelial tissue changes after prolonged and excessive vibration exposure. The present study demonstrates that epithelial surface changes can be measured objectively to assess the severity of vocal fold epithelial surface damage. Future studies are planned to determine at what point tissue damage can and cannot be reversed and/or restored. These studies will be helpful in the development of guidelines and trajectories for normal tissue recovery.

In summary, this study represents the first attempt to quantify the degree of vocal fold epithelial surface damage after acute episodes of phonotrauma. Electron microscopy and advanced imaging measurement techniques were used to determine the effects of increased time- and magnitude-doses of vibration exposure on the vocal fold epithelial surface. Results of the present study revealed a significant decrease in microprojection density, microprojection height, and depth of the remaining cell surface layer with increasing time and magnitudes of vibration exposure. We hypothesize that destruction to the vocal fold epithelial surface and its crucial functions, such as water and metabolic product movement across the cell membrane and stabilization of surface secretions, may provide insight into the pathophysiology of normal and diseased cellular physiology and vocal fold tissue function. The present study provides an important foundation for future studies of long-term tissue recovery from phonotrauma.
